# Exosomal circRNAs: biogenesis, effect and application in human diseases

**DOI:** 10.1186/s12943-019-1041-z

**Published:** 2019-07-05

**Authors:** Yangxia Wang, Jinbo Liu, Junfen Ma, Ting Sun, Quanbo Zhou, Weiwei Wang, Guixian Wang, Pingjun Wu, Haijiang Wang, Li Jiang, Weitang Yuan, Zhenqiang Sun, Liang Ming

**Affiliations:** 1 Key Clinical Laboratory of Henan Province, Department of Clinical Laboratory, The First Affiliated Hospital of Zhengzhou University, Zhengzhou, 450052 Henan China; 2 Department of Colorectal Surgery, The First Affiliated Hospital of Zhengzhou University, Zhengzhou, 450052 Henan China; 3 Department of Gastrointestinal Surgery, Affiliated Tumor Hospital, Xinjiang Medical University, Ürümqi, 830011 Xinjiang China; 4 Department of Pathology, The First Affiliated Hospital, Zhengzhou University, Zhengzhou, 450052 Henan China; 5 Department of Radiotherapy, The First Affiliated Hospital, Zhengzhou University, Zhengzhou, 450052 Henan China; 6 Department of Cardiac surgery, The Third People’s Hospital of Henan Province, Zhengzhou, 450000 Henan China

**Keywords:** Exosome, CircRNA, Exosomal circRNA, Tumor microenvironment, Biomarker

## Abstract

Exosomes have emerged as critical mediators of intercellular communication, both locally and systemically, by regulating a diverse range of biological processes between cells. Circular RNA (circRNA) is a novel member of endogenous noncoding RNAs with widespread distribution and diverse cellular functions. Recently, circular RNAs have been identified for their enrichment and stability in exosomes. In this review, we outline the origin, biogenesis and function of exosomal circRNAs as well as their roles in various diseases. Although their precise roles and mechanisms of gene regulation remain largely elusive, exosomal circRNAs have potential applications as disease biomarkers and novel therapeutic targets.

## Background

In the past 30 years, one of the most revolutionary contributions to cell biology was the discovery of exosomes; exosomes are nanoscale (30–150 nm) extracellular vesicles of endocytic origin that are shed by most types of cells and circulate in bodily fluids such as blood, urine, saliva, and breast milk [1]. Exosomal contents have been shown to be broad, composed of various growth factors, proteins, lipids, and nucleic acids, long noncoding RNAs and circular RNAs (circRNAs) [2]. Exosomes are intraluminal vesicles generated within the endolysosomal system and secreted by the fusion of multivesicular endosomes (MVEs), which are shed from the plasma membrane [3]. CircRNAs get its closed loop structures from back-spliced exons in nucleus. After transferring into cytoplasm, circRNAs can not only bind with miRNAs or protein to exert various functions, but can be sorted into MVEs along with an abundant cargo of other nucleic acids, lipid and proteins [4]. Then exosomes are secreted from the parent cells into bodily fluid, through which circRNAs start their circulation and activate their biological functions.

After released into the extracellular environment, exosomes can reach recipient cells and deliver their cargos (like circRNAs) to elicit functional responses and induce a series of phenotypic changes. Some groups reported several mediators of specific interactions contribute to exosomes to find their target cells, including integrins, lipids, lectins, and extracellular matrix (ECM). For example, exosomes-derived from cancer cells can be targeted to specific organs, such as liver and lung to promote the formation of premetastatic niche dependent on their integrin composition [5]. Additionally, membrane vesicles of cellular origin expose adhesion molecular on their surface, which could favor their capture by target cells [6].

Importantly, an increasing number of studies have focused on understanding the function of exosomes in mediating intercellular communication, tumor microenvironment, immune system functions [7–9], development and differentiation, cell signaling and viral replication [10]. Not only are exosomes reported in practical applications, they are also utilized for clinical diagnostics and therapeutic development. To summarize, exosomes are being intensively investigated as a valuable source of novel biomarkers due to the specific cargo loaded by their progenitor cells.

Circular RNAs, a novel class of endogenous noncoding RNAs, are characterized by their covalently closed loop structures without 5′ caps and 3′ poly tails. They were first proposed in RNA (ribonucleic acid) viruses as a viroid in 1976 and were thought to be a result of splicing errors for several decades [11]. Currently, with the development of deep RNA sequencing (RNA-seq) technologies and novel bioinformatic approaches, abundant and diverse circRNAs have been detected and identified, including few circRNA functions, such as regulating transcription in the nucleus [12, 13], functioning as efficient microRNA sponges [14–16], competing with pre-mRNA splicing [17], and serving as circRNA-protein interactions [18]. Additionally, recent studies have shown that circRNAs are abundantly present in the hematopoietic compartment [19] and participate in neuronal development [20, 21]; some circRNAs are reported to be associated with the initiation, progression and metastasis of tumors [22–24]. In addition to these encouraging advances, it is worthwhile to note that their high abundance, relative stability, and evolutionary conservation among species endow circRNAs with utility as potential biomarkers for many diseases, especially for cancers [25, 26].

Interestingly, recent studies have shown that circRNAs are enriched and stable in exosomes, which can be detected in the circulation and urine [27]. For instance, it was reported that circRNAs in gastric cancer (GC) could be transferred to recipient cells via exosome delivery, suggesting that exosomal circRNAs play significant roles in the invasion and peritoneal metastasis of GC [28]. Moreover, exosomal circRNAs were also found in platelet-derived extracellular vesicles [29], hepatic cells [30] and pancreatic cancer cells [31]. Studies have reported that it is possible that cells could transfer circRNAs by excreting them in exosomes. Because exosomes can be received by many types of cells, including macrophages, they may also act as messengers in cell-to-cell communications. Interestingly, some other studies have concluded that the clearance of intracellular circRNAs was associated with exosomes, and exosomes themselves could be further removed by the reticuloendothelial system or secreted by the kidneys and liver [32, 33].

In this review, we briefly address the significance and role of exosomes as intercellular communication cargo in various diseases, including tumors, neurodegenerative disease, infections and autoimmune disorders, by transferring DNA, RNA, proteins and lipids, with a focus on circRNAs. Recent advances in exosome-derived circRNA isolation will also be discussed along with their major technical challenges. Particularly, we summarize the role of exosomal circRNAs in the formation of some diseases along with their biological functions, with an emphasis on their potential as promising diagnostic molecular markers and therapeutic targets.

### Emerging roles of exosome in physiological and pathological status

Notably, it is increasingly evident that exosomes play an important role not only on the regulation of normal physiological status, such as tissue regeneration [34], immune surveillance [35], blood circulation [36] and stem cell plasticity [37], but also in the pathological conditions in several diseases. For example, exosomes take participate in tissue repair via delivering specific cargoes to the recipient cells. They can enhance the function of regulatory T cells and suppress the CD8^+^ cell and natural killer (NK) activity. In the blood circulation, exosome also take part in breast cancer with lung metastasis from docking Nano platform [38]. Exosomes have been shown in converting the hematopoietic stem cells (HSCs) phenotype into liver cells phenotype [39].

Apart from their fundamental roles in physiological condition, exosomes have a pivotal role in disease pathogenesis, especially in tumor progression. For example, the activated PMN (polymorphonuclear leukocyte) exosomes could bind and degrade extracellular matrix (ECM) by the integrin Mac-1 and NE, respectively, inducing the disorders of chronic obstructive pulmonary disease (COPD) and bronchopulmonary dysplasis (BPD) [40]. A key study showing the clinical relevance and linkage of exosome to disease was reported in 2012 by Wan J and colleagues. This study represented that exosome core component gene was responsible for pontocerebellar hypoplasia and spinal motor neuron degeneration.

### Roles of circRNAs in normal physiology and pathological status

Some circRNAs are extraordinarily enriched in the mammalian brain [26] and preferentially back-spliced in neuronal cell lines [41], suggesting that circRNAs have a potential to regulate synaptic function. Similarly, many circRNAs are significantly enriched in synaptogenesis than their linear isoforms during central nervous system aging [42]. In addition, circRNAs are associated with disease pathologic processes. For instance, circRNAs act as microRNA (miRNA) sponges to modulate gene expression. For example, circRNAs are relatively abundant in the brain [26] and accumulate to a high level during the central nervous system aging [42]. Specifically, the circRNA ciRS-7 (also termed CDR1as), which harbors more than 70 conventional miR-7-binding locations, has been observed as a miRNA inhibitor [43]. Additionally, circNT5E acts as a sponge that directly binds miR-422a and inhibits miR-42an activity, regulating multiple pathologic processes, including cell proliferation, migration and invasion in glioblastoma tumorigenesis [14]. Although circRNAs are generally expressed in small quantities, numerous studies have shown that circRNAs are responsible for physiological development and different diseases, such as cardiovascular diseases [44–47], neurological disorders [20, 48, 49], and tumors [50–52]. Currently, circRNAs have also been reported to be enriched and stable in saliva [53], plasma [54], and even in serum exosomes [4], suggesting the potential of circRNAs as responsive biomarkers.

### Origin of exosomal circRNAs

Generally, almost all cell types release exosomes that are enriched in plasma as well as other bodily fluids, including saliva, urine, semen, sputum and breast milk. Exosomes are also generated by both immune and nonimmune cells that have a significant role in the regulation of immunity [55]. Moreover, exosome release has been suggested to have prognostic relevance; increased levels of serum exosomes typically predicts unfavorable outcomes [56, 57]. Notably, Li first reported the enrichment and stability of abundant circRNAs in exosomes compared to the producer cells by using RNA-sequence analyses [58]. Although research on exosomal circRNAs is currently gaining momentum, the functions and characteristics of exosomal circRNAs remain largely unclear.

The composition of exosomal circRNAs may be modulated by changes in associated miRNA levels in donor cells, and subsequently this molecular information is transferred to recipient cells. In serum exosomes, circRNAs also contribute to the onset and progression of many neoplasias through RNA-RNA competitive interactions. For instance, circHIPK3 and TUG1 were upregulated, while LncRNA UCA1 was downregulated via RNA silencing, mitogen-activated protein kinase (MAPK) inhibition and in silico analyses [59]. Studies have shown that circRNAs are distinctly downregulated in a KRAS mutant and can be transferred to the exosomes of colorectal cancer cell lines [60]. Currently, exosomal circ-PDE8A was reported to be associated with tumor progression and lymphatic invasion by the miR-338/MACC1/MET pathway in pancreatic ductal adenocarcinoma [61]. Moreover, exosomal circRNA has been observed to promote white adipose browning via targeting the miR-133/PRDM16 pathway, providing a novel perspective into our understanding of cancer-associated cachexia [62]. Additionally, exosomal circRNAs originate from endometrial cancer and hepatocellular carcinoma by targeting the deubiquitination-related USP7, promoting tumor growth [63].

**Fig. 1:**
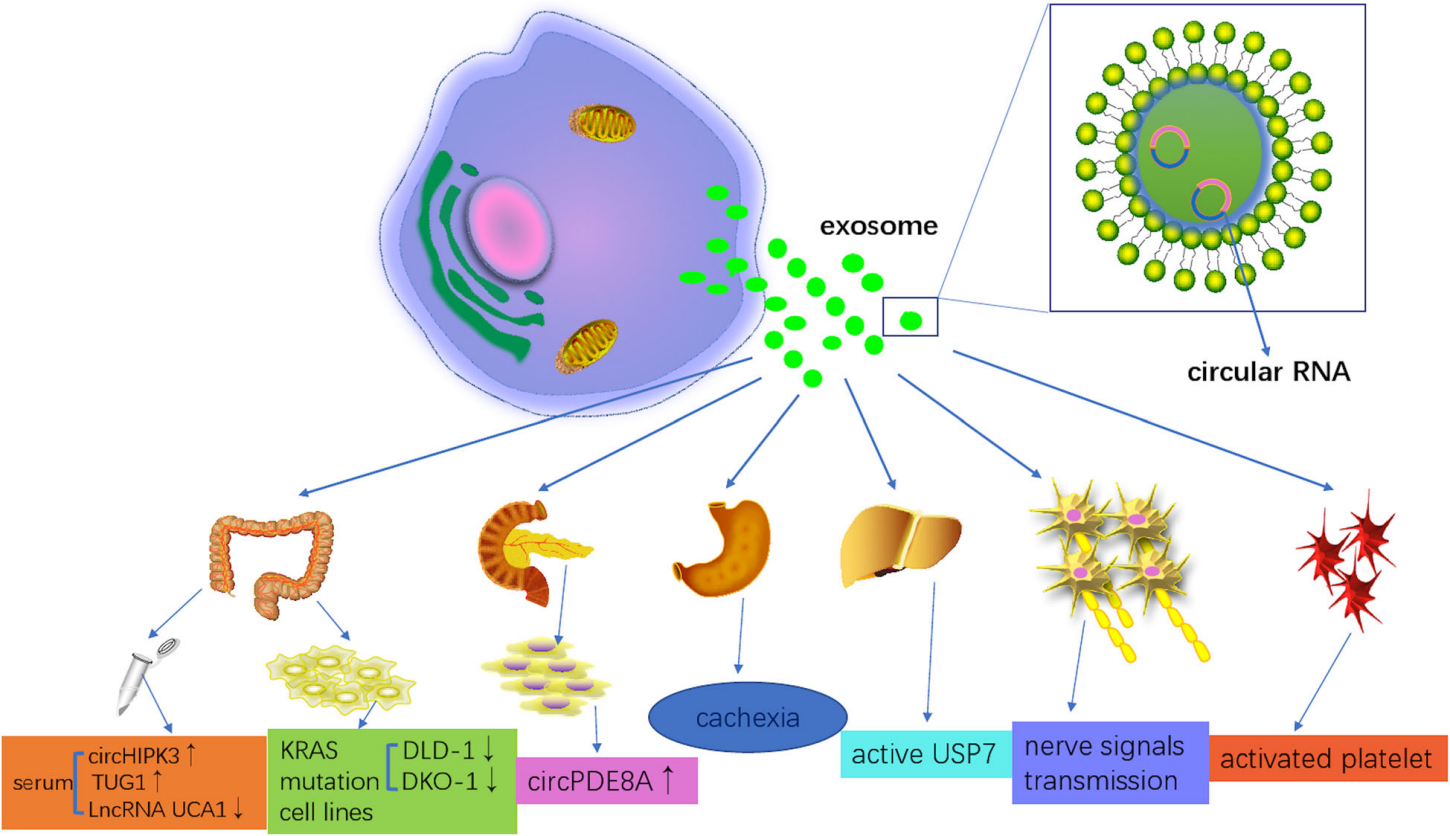
Origin and biogenesis of exosomal circRNAs in various diseases. Most cells secrete exosomes under different physiological and pathophysiological conditions. CircRNAs can be transferred through exosomes between donor cells and recipient cells as a messenger to mediate multiple signaling pathways

Apart from cancer origin, exosomal circRNAs commonly originate from activated human platelets, which are associated more with hemostasis, inflammation and wound healing than other hematopoietic cells [29, 64, 65]. Interestingly, Zhao showed that exosomal circRNAs might be responsible for the growth and repair of neurons, the transmission of nerve signals, and the regulation of important signaling pathways, specifically in glutamatergic synapses and the cGMP-PKG signaling pathway [66] (Fig. 1). Consequently, further research on exosomal circRNAs not only has profound impacts on understanding the functions and characteristics of exosomes but also provides a novel avenue for many disease diagnoses and targeted Therapies.

### Biological functions of exosomal circRNAs

Exosome plays a key factor in the formation of premetastatic niches [67] and tumor microenvironment [68]. Additionally, exosomes have been found to associated with tumor cell survival and tumor recurrence through mediating immune suppression and immune surveillance [69, 70]. Interestingly, some circRNAs can be detected in exosomes derived from serum, urine and tumor, exosomal circRNAs may participate in the processes of cell growth, angiogenesis, epithelial mesenchymal transition, and targeted therapy. In this section, we focus on the essential roles of exosomal circRNAs in cell proliferation, tumor metastasis, and drug resistance.

### Exosomal circRNAs and proliferation

Deregulated proliferation is one of the most important factors in neoplastic transformation; thus, increasing attention has been given to the understanding of the mechanisms of cell cycle regulation [71]. In previous reports, miRNAs were associated with the regulation of the cell cycle and the proliferation of hepatocellular carcinoma (HCC) cells [72, 73]. Interestingly, for arsenite-transformed HaCat cells, Dai reported that exosomal circRNA_100284 regulates the cell cycle by acting as a sponge of miR-217 [74] and inhibits cell proliferation by inducing arrest in the G2/M phase of the cell cycle and targets EZH2 in various cancers [75, 76], including HCCs [77, 78]. However, the underlying mechanisms of the regulation of EZH2 via miR-217 and how it affects malignant transformation still require further characterization.

**Fig. 2:**
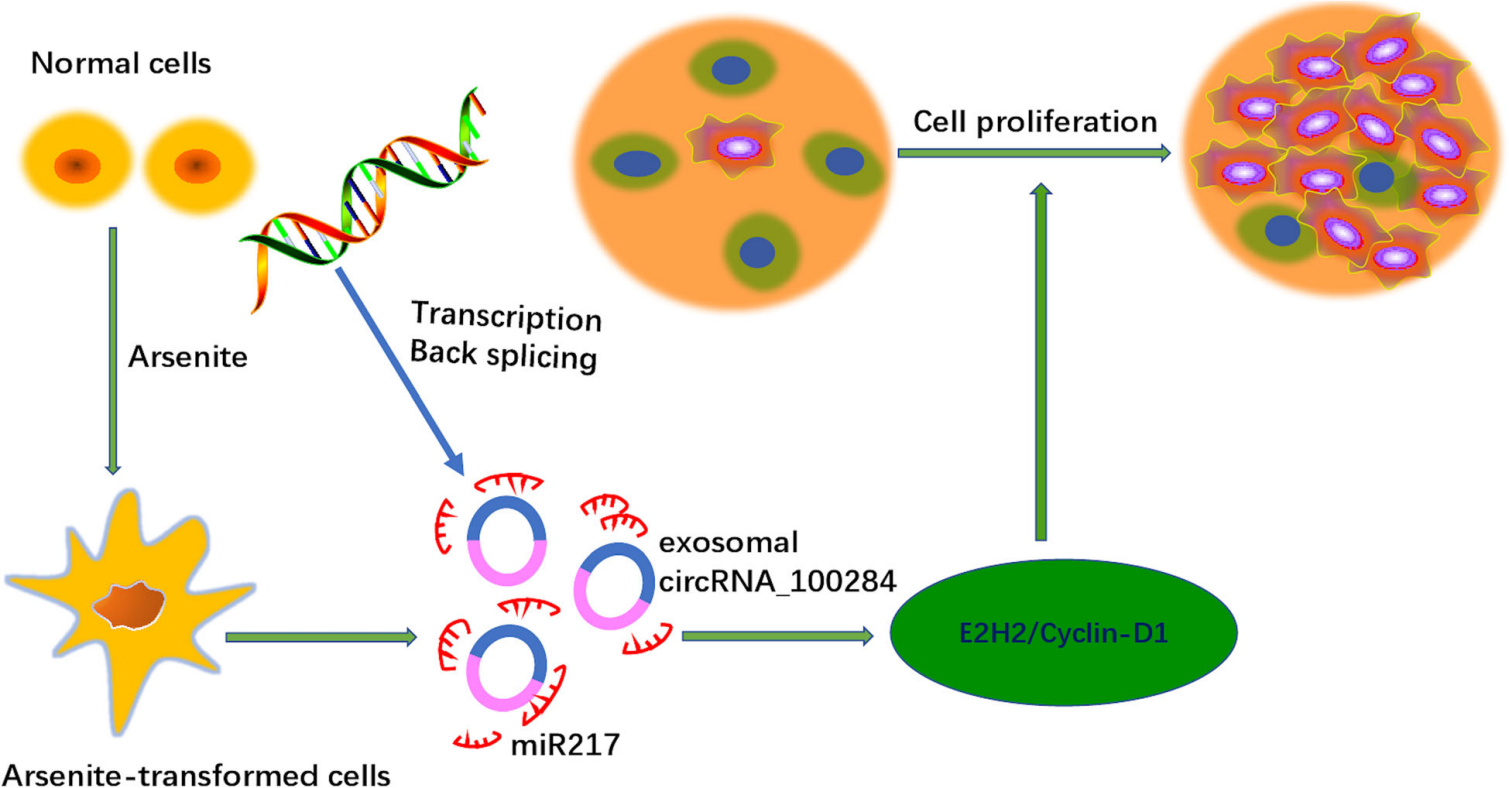
Schematic illustration of the role of exosomal circRNAs in cell proliferation. Chronic exposure to arsenite increases exosomal circRNA_100284 levels, which accelerate the cell cycle and promote proliferation by acting on miR217. E2H2 and cyclin D1, promising biomarkers of proliferation, are extensively activated to regulate the G1 to S phase transition, thus inducing cell proliferation

Notably, recent studies have shown that the levels of circRNAs were increased in exosomes secreted from arsenite-transformed cells. The overexpression of miR-217 in normal cells reduced the expression of EZH2 (a promising biomarker of proliferation) [79, 80] and clclin-D1, which regulates the G1 to S phase transition in the cell cycle [81] (Fig. 2). This evidence suggests that exosomal circRNAs induced an accelerated cell cycle and promoted the proliferation of normal cells. Additionally, exosomal circRNAs secreted from adipose tissues can regulate the deubiquitination in HCC. Moreover, studies in vivo proved that overexpressed circ-deubiquitination (circ-DB) significantly downregulated miR-34a, leading to the activation of miR-34a/USP7/CyclinA2 signaling pathway. Consequently, exosomal circRNAs promote cell growth and suppress damage to DNA [63].

Intriguingly, a recent study demonstrated that exosomal circRASSF2 promoted laryngeal squamous cell carcinoma (LSCC) progression. Researchers have found circRASSF2 expression was significantly and consistently increased in LSCC tumor tissues compared with control groups [82]. Moreover, they knockdown exosomal circ-RASSF2 remarkably inhibited cell proliferation and migration via miR-302b-3p/IGF-1R axis, indicating the significance of exosomal circRNAs in tumor cell proliferation.

### Exosomal circRNAs and metastasis

**Fig. 3:**
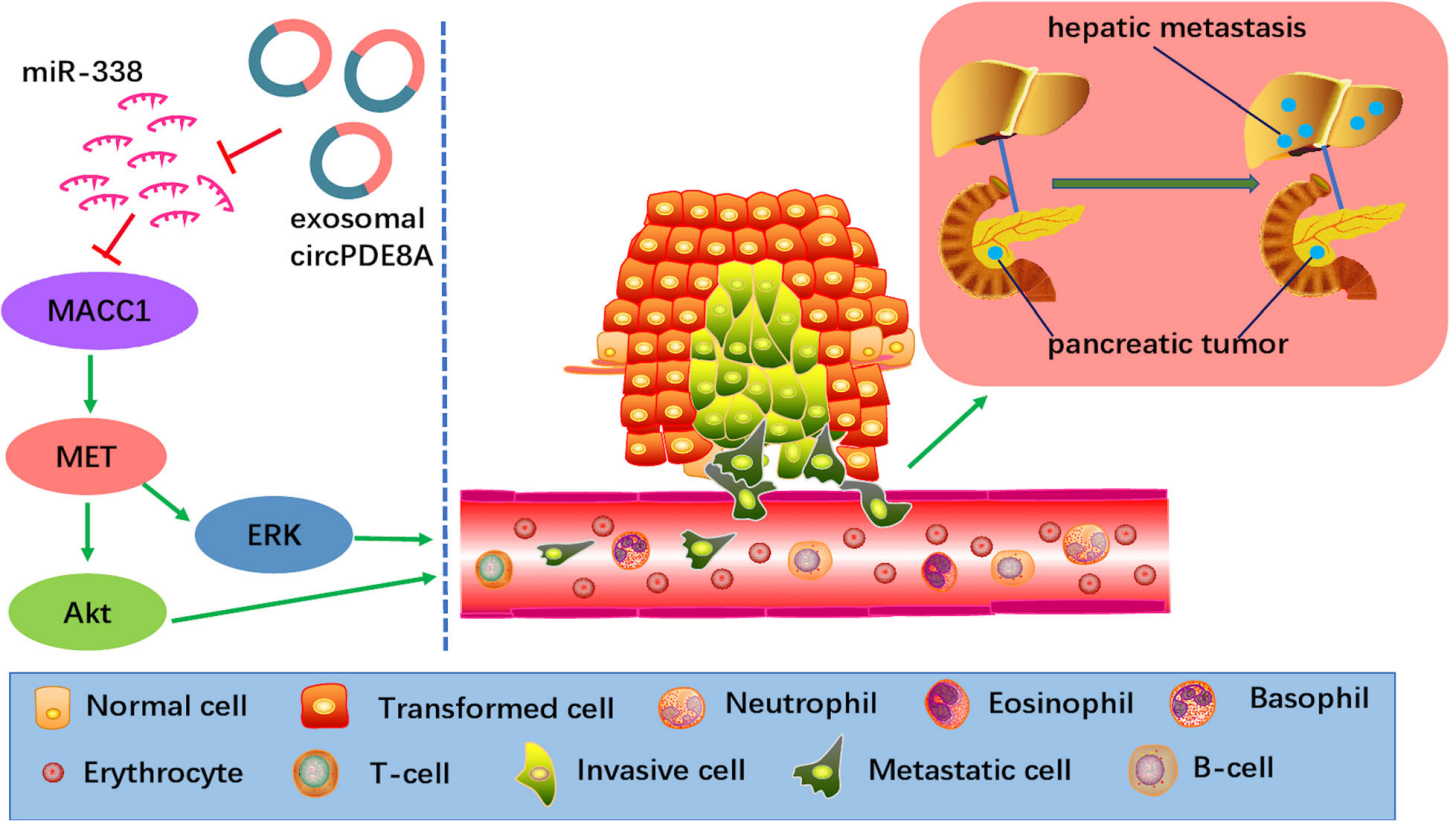
The role of exosomal circRNAs in metastasis. Exosomal circPDE8A acted as a competing endogenous RNA sponge with miR-338 and induced invasive growth via the MACC1, MET, ERK, and AKT pathways. Eventually, this process led to an increase in vascular endothelial permeability and promoted pancreatic tumor hepatic metastasis

Overwhelming studies conformed that tumor onset and metastasis are associated with many oncogenes and that many oncogenic pathways are involved [83]. Nowadays, growing evidences indicate that cancer cell-to-cell communication and surrounding stroma contribute to metastasis. For example, Li et al. first identified that an important role of exosomal circ-PED8A in pancreatic ductal adenocarcinoma (PDAC). They found that a high expression of exosomal circ-PED8A in PDAC tissue was associated with lymphatic invasion, TNM stage and a poor survival rate. Further research revealed that circ-PDE8A promoted tumore cells growth via upregulating MET, which is a tyrosine kinase receptor, one of the key oncogenes for a subset of epithelial tumors, including PDAC [84]. Moreover, tumor-excreted circ-PDE8A could be released into blood circulation by exosome transportation, acting as a ceRNA for miR-338 to regulate MACC1 and promote invasive metastasis through the MACC/MET/ERK or AKT pathways (Fig. 3). Similarly, a recent study confirmed that circNRIP1 could be transmitted by exosome connection between gastric cancer (GC) cells, and exosomal circNRIP1 sponges miR-149-5p to affect the AKT1/mTOR signaling pathway and to promote the proliferation, migration and metastasis in gastric cancer [85]. Similarly, this phenomenon was also identified in breast cancer cells and patients [86]. These novel findings indicated the significance role of exosomal circRNAs in tumor metastasis.

Apart from the mentioned above, emerging evidence shows that the expression of certain exosomal circRNAs play important roles in the progression and metastasis of Hepatocellular carcinoma (HCC). For instance, Wang recently proved that exosomal circPTGR1 contributes to hepatocellular carcinoma metastasis [87]. Researchers found the high expression of exosomal circPTGR1 was related to the clinical stage and prognosis. In further, knockdown of exosomal circPTGR1 expression significantly suppressed tumor invasion and migration in non- and low-metastatic cell lines. According to bioinformatics analysis and both in vivo and in vitro experiments, exosomal circPTGR1 competed with MET interactions to target miR449a, leading to the dysregulation of tumor microenvironment and the promotion of HCC development.

Pathological epithelial mesenchymal transition (EMT) plays essential roles in tumor progression and the initiation of metastasis. Recently, Jie found that circ-IARS, secreted by pancreatic cancer cells and located in exosomes, was extensively expressed in pancreatic cancer tissues, and its expression level was responsible for tumor vasculature, tumor node metastasis (TNM) stage and liver metastasis [31]. Cancer cells must cross the endothelial barrier during extravasation for tumor distant metastasis [88]. Interestingly, the Ras homolog gene family, member A (RhoA), is associated with cytoskeleton regulation and reduces the expression of the junction ligand protein Zonula occludens-1 (ZO-1), leading to endothelial barrier dysfunction and endothelial solute permeability [89–91]. It was indicated that the tumor suppressor miR-122 incudes mesenchymal-epithelial transition and inhibits the migration and metastasis of HCC by targeting RhoA [92]. Moreover, increased activity and expression of RhoA in human microvascular vein endothelial cells (HUVECs) was recently indicated to increase F-actin expression and decrease ZO-1 [93, 94], which damages the endothelial barrier function [89–91] and reinforces endothelial permeability [95–99]. In addition, Chen X et al. revealed that circPRMT5 was up-regulated in serum and urine exosomes from urothelial carcinoma of the bladder (UCB) tissues [100]. Further study confirmed exosomal circPRMT5 promoted UCB cell EMT via blocking with miR-30c, leading to the high expression of SNAIL1 and E-cadherin. Consequently, circRNAs can be transferred through exosomes from cancer cells to other cells as messengers, mediating cell-to-cell communication, regulating endothelial permeability, and thus promoting tumor metastasis.

### Exosomal circRNAs and drug resistance

Drug resistance is considered an enormous impediment in the treatment of cancer patients. As various underlying mechanisms are related to the progression of drug resistance, exosomes have attracted and gained universal attention as a novel therapeutic avenue [101, 102]. Importantly, exosomes transmit multi-drug resistance (MDR)-associated proteins [103] and noncoding RNAs to target cells [104], including circRNAs. For instance, Li L. et al. currently revealed serum exosomal circRNA (FECR1) was aberrantly upregulated in small cell lung cancer (SCLC) compared with control groups. In addition, serum exosomal- FECR1 was asscociated with clinical chemotherapy and poor survival in SCLC patients [105]. These evidences suggested a possible relationship between exosomal circRNAs dysregulation and drug resistance.

Accumulating evidence has shown that epithelial cancers, such as colorectal cancer (CRC), are preferentially resistant to many advanced drug therapies. Epidermal growth factor receptor (EGFR), a member of the HER-ErbB family, plays a significant role in the proliferation, angiogenesis, gene mutation and drug resistance in CRC. KRAS is an intracellular signaling molecule, and its mutations are found in approximately 40% of colorectal tumors [106]. Therefore, the identification of the key signaling pathways impacted by the KRAS mutation is beneficial for our understanding of how to pharmacologically target therapies.

Interestingly, the abundance of circRTN4 was remarkably upregulated in the exosomes derived from DLD-1 CRC cells, which possess a KRAS mutation compared with DKs-8 cells (wild-type KRAS). However, the intracellular abundance of circRTN4 was significantly downregulated in DLD-1 and DKO-1 cells, indicating exosomal circRNAs are independent of intracellular circRNA and may be actively transmitted between exosomes and the cytoplasm [60, 107]. These findings provide new insight into the function of exosomal circRNAs and demonstrate their ability to regulate chemotherapy resistance.

### Clinical applications

The nascent era of the clinical application of exosomel circRNA is growing rapidly. Emerging evidences suggest that exosomal circRNAs are released from various cells and carry signaling molecules for cellular communication, even the regulation of organ function. In addition, the expression profiles of exosomal circRNAs in patients distinguish from healthy groups, it is possible that exosomal circRNAs can function as molecular markers of some tumors to support diagnosis. Exosomal circRNAs are also being recognized as crucial mediators of healthy and diseased states and may become promising biomarkers of specific conditions. For instance, the levels of exosomal circRNAs were significantly up-regulated in DKs-8 cells compared to DLD-1 and DKO-1cells [60], suggesting that exosomal circRNAs may serve as a promising biomarker in colon cancers. Similarly, exosomal circular RNA IARS expression was higher than those of control groups both in pancreatic cancer tissues and in plasma exosomes [31], the result of this study indicated that the existence of exosomal circRNA could be a useful marker of PDAC diagnosis and prognostic prediction.

In addition, exosome-derived miRNA and proteomic materials may be used as diagnostic indicators of various tumors, such as ovarian, prostate and lung cancer [108–110]. Unlike miRNA, circRNAs are exceptionally stable, conserved and have cell−/tissue-specific expression patterns, which suggest their potential applications as gene regulators as well as their possibility as molecular diagnostic and prognostic biomarkers. Importantly, the unique cellular stability and function of circRNAs to sponge miRNA and proteins may also indicate that circRNA is a promising vehicle for targeted drug delivery [111]. Despite its large potential, our understanding of exosomal circRNA is still in infancy.

Intriguingly, according to Li, 1215 circRNAs were identified in the human serum exosomes, and more than 90% of the detected circRNAs consisted of exons showed high stability and conserved exonuclease cleavage, suggesting that circRNAs can be actively transferred from cells to exosomes and indicating the underlying possibility of tumor diagnostic markers. Due to their applicability, specificity and accessibility, exosomal circRNAs have the potential for cellular therapy and for the theoretical concerns of neoplastic transformation from peripheric blood-, tissue-, or bone marrow derived stem cell transplantation.

## Conclusions and perspectives

Exosomal circRNAs are a novel frontier in cancer research, and only a few of circRNAs have established functional roles or clinical applications. Exosomal circRNAs modulate cellular proliferation, invasion, migration, metastasis and drug resistance. In addition, exploring the mysterious connection of exosome and circRNA may provide a vital hint to understand the biological functions of exosomal circRNAs. Currently, there are two assumptions of circRNA in exosomes. Interestingly, exosome might be a double-edged sword for circRNA. One is that exosome is a communication messenger between cells due to its accessibility in bodily fluids. Exosome can carry circRNA to transfer biological information and material to target cells and protect circRNA from clearance. The other is that exosome could reduce the accumulation of circRNAs and help circRNA clearance. Erika Lasda point out that cells can eliminate circRNA by excreting them via extracellular vesicles [32], which could be taken up by other specific cells, including macrophages.

Despite the abovementioned encouraging advances, challenges and difficulties of exosomal circRNAs in clinical applications exist in multiple aspects. First, due to their low abundance, circRNAs are difficult to be detected in exosomes with an accurate approach and algorithms. Second, the circular conformation and sequence overlap with linear mRNA counterparts have made the precise evaluation of circRNA expression and function challenging. It is worthy nothing that these problems will be solved with the advanced technology, improved experimental approaches and further research.

Recent studies have indicated that exosomal circRNAs may have a significant influence on pathophysiologic processes. The connection of exosomal circRNAs with cancer has become a popular research field. However, there are still some questions that need to be addressed. First, we lack an understanding of how circRNAs are ultimately degraded. In addition, the mechanism of how circRNAs are enriched during exosome formation is unknown. One possibility is that circRNAs are abundant in the cytoplasm and passively included in exosomes during their formation. Alternatively, circRNAs may be actively transferred from the cytoplasm into exosomes. Future studies investigating exosomal circRNAs in the hematopoietic system, immune response, nervous disorders, cancer development and other biological settings and diseases will further unveil the mystery of exosomal circRNAs. Therefore, revealing cancer pathogenesis mechanisms and seeking novel potential diagnostic biomarkers or therapeutic targets will be popular topics in the future.

## Data Availability

Not applicable.

## Abbreviations

BPD: Bronchopulmonary dysplasis
ceRNA: competing endogenous RNA
circ-DB: circ-deubiquitination
CircRNA: Circular RNA
COPD: Chronic obstructive pulmonary disease
CRC: Colorectal cancer
ECM: Extracellular matrix
EGFR: Epidermal growth factor receptor
GC: Gastric cancer
gDNA: genomic DNA
HCC: Hepatocellular carcinoma
HSCs: Hematopoietic stem cells
HUVECs: RhoA in human microvascular vein endothelial
LSCC: Laryngeal squamous cell carcinoma
MAPK: Mitogen-activated protein kinase
MDR: Multi-drug resistance
MET: Mesenchymal to epithelial
miRNAs: microRNAs
mRNA: messenger RNA
MVB: Multivesicular body
MVEs: Multivesicular endosomes
PDAC: Pancreatic ductal adenocarcinoma
RNA: Ribonucleic acid
RNA-seq: RNA sequencing
SCLC: Small cell lung cancer
TNM: Tumor node metastasis
ZO-1: Zonula occludens-1

## References

[1] Boriachek Kseniia, Islam Md. Nazmul, Möller Andreas, Salomon Carlos, Nguyen Nam-Trung, Hossain Md. Shahriar A., Yamauchi Yusuke, Shiddiky Muhammad J. A. (2017). Biological Functions and Current Advances in Isolation and Detection Strategies for Exosome Nanovesicles. Small.

[2] Braicu C, Tomuleasa C, Monroig P, Cucuianu A, Berindan-Neagoe I, Calin GA (2015). Exosomes as divine messengers: are they the Hermes of modern molecular oncology?. Cell Death Differ.

[3] Raposo G, Stoorvogel W (2013). Extracellular vesicles: exosomes, microvesicles, and friends. J Cell Biol.

[4] Bao C, Lyu D, Huang S (2016). Circular RNA expands its territory. Mol Cell Oncol.

[5] Hoshino A, Costa-Silva B, Shen TL, Rodrigues G, Hashimoto A, Tesic Mark M, Molina H, Kohsaka S, Di Giannatale A, Ceder S (2015). Tumour exosome integrins determine organotropic metastasis. Nature.

[6] Thery C, Ostrowski M, Segura E (2009). Membrane vesicles as conveyors of immune responses. Nat Rev Immunol.

[7] Yu G, Jung H, Kang YY, Mok H (2018). Comparative evaluation of cell- and serum-derived exosomes to deliver immune stimulators to lymph nodes. Biomaterials.

[8] Zhang Y, Wang XF (2015). A niche role for cancer exosomes in metastasis. Nat Cell Biol.

[9] Gao L, Wang L, Dai T, Jin K, Zhang Z, Wang S, Xie F, Fang P, Yang B, Huang H (2018). Tumor-derived exosomes antagonize innate antiviral immunity. Nat Immunol.

[10] Alenquer M, Amorim MJ (2015). Exosome biogenesis, regulation, and function in viral infection. Viruses.

[11] Sanger HL, Klotz G, Riesner D, Gross HJ, Kleinschmidt AK (1976). Viroids are single-stranded covalently closed circular RNA molecules existing as highly base-paired rod-like structures. Proc Natl Acad Sci U S A.

[12] Li Z, Huang C, Bao C, Chen L, Lin M, Wang X, Zhong G, Yu B, Hu W, Dai L (2017). Corrigendum: exon-intron circular RNAs regulate transcription in the nucleus. Nat Struct Mol Biol.

[13] Li Z, Huang C, Bao C, Chen L, Lin M, Wang X, Zhong G, Yu B, Hu W, Dai L (2015). Exon-intron circular RNAs regulate transcription in the nucleus. Nat Struct Mol Biol.

[14] Wang R, Zhang S, Chen X, Li N, Li J, Jia R, Pan Y, Liang H (2018). CircNT5E acts as a sponge of miR-422a to promote glioblastoma tumorigenesis. Cancer Res.

[15] Granados-Riveron JT, Aquino-Jarquin G (1859). The complexity of the translation ability of circRNAs. Biochim Biophys Acta.

[16] Qian L, Yu S, Chen Z, Meng Z, Huang S, Wang P (1870). The emerging role of circRNAs and their clinical significance in human cancers. Biochim Biophys Acta Rev Cancer.

[17] Ashwal-Fluss R, Meyer M, Pamudurti NR, Ivanov A, Bartok O, Hanan M, Evantal N, Memczak S, Rajewsky N, Kadener S (2014). circRNA biogenesis competes with pre-mRNA splicing. Mol Cell.

[18] Du WW, Zhang C, Yang W, Yong T, Awan FM, Yang BB (2017). Identifying and characterizing circRNA-protein interaction. Theranostics.

[19] Bonizzato A, Gaffo E, Te Kronnie G, Bortoluzzi S (2016). CircRNAs in hematopoiesis and hematological malignancies. Blood Cancer J.

[20] Floris G, Zhang L, Follesa P, Sun T (2017). Regulatory role of circular RNAs and neurological disorders. Mol Neurobiol.

[21] Chen W, Schuman E (2016). Circular RNAs in brain and other tissues: a functional enigma. Trends Neurosci.

[22] Hansen TB, Kjems J, Damgaard CK (2013). Circular RNA and miR-7 in cancer. Cancer Res.

[23] Guarnerio J, Bezzi M, Jeong JC, Paffenholz SV, Berry K, Naldini MM, Lo-Coco F, Tay Y, Beck AH, Pandolfi PP (2016). Oncogenic role of fusion-circRNAs derived from Cancer-associated chromosomal translocations. Cell.

[24] Yu J, Xu QG, Wang ZG, Yang Y, Zhang L, Ma JZ, Sun SH, Yang F, Zhou WP (2018). Circular RNA cSMARCA5 inhibits growth and metastasis in hepatocellular carcinoma. J Hepatol.

[25] Han D, Li J, Wang H, Su X, Hou J, Gu Y, Qian C, Lin Y, Liu X, Huang M (2017). Circular RNA circMTO1 acts as the sponge of microRNA-9 to suppress hepatocellular carcinoma progression. Hepatology.

[26] Rybak-Wolf A, Stottmeister C, Glazar P, Jens M, Pino N, Giusti S, Hanan M, Behm M, Bartok O, Ashwal-Fluss R (2015). Circular RNAs in the mammalian brain are highly abundant, conserved, and dynamically expressed. Mol Cell.

[27] Reg GL, Zhu J, Li J, Meng XM. Noncoding RNAs in acute kidney injury. J Cell Physiol. 2019;234:2266–76. 10.1002/jcp.27203 30146769

[28] Yan Y, Fu G, Ye Y, Ming L (2017). Exosomes participate in the carcinogenesis and the malignant behavior of gastric cancer. Scand J Gastroenterol.

[29] Preusser C, Hung LH, Schneider T, Schreiner S, Hardt M, Moebus A, Santoso S, Bindereif A (2018). Selective release of circRNAs in platelet-derived extracellular vesicles. J Extracell Vesicles.

[30] Dai X, Chen C, Yang Q, Xue J, Chen X, Sun B, Luo F, Liu X, Xiao T, Xu H (2018). Exosomal circRNA_100284 from arsenite-transformed cells, via microRNA-217 regulation of EZH2, is involved in the malignant transformation of human hepatic cells by accelerating the cell cycle and promoting cell proliferation. Cell Death Dis.

[31] Li J, Li Z, Jiang P, Peng M, Zhang X, Chen K, Liu H, Bi H, Liu X, Li X (2018). Circular RNA IARS (circ-IARS) secreted by pancreatic cancer cells and located within exosomes regulates endothelial monolayer permeability to promote tumor metastasis. J Exp Clin Cancer Res.

[32] Lasda E, Parker R (2016). Circular RNAs co-precipitate with extracellular vesicles: a possible mechanism for circRNA clearance. PLoS One.

[33] Choi H, Lee DS (2016). Illuminating the physiology of extracellular vesicles. Stem Cell Res Ther.

[34] Bjorge IM, Kim SY, Mano JF, Kalionis B, Chrzanowski W (2017). Extracellular vesicles, exosomes and shedding vesicles in regenerative medicine - a new paradigm for tissue repair. Biomater Sci.

[35] Plebanek MP, Angeloni NL, Vinokour E, Li J, Henkin A, Martinez-Marin D, Filleur S, Bhowmick R, Henkin J, Miller SD (2017). Pre-metastatic cancer exosomes induce immune surveillance by patrolling monocytes at the metastatic niche. Nat Commun.

[36] Yamashita T, Takahashi Y, Nishikawa M, Takakura Y (2016). Effect of exosome isolation methods on physicochemical properties of exosomes and clearance of exosomes from the blood circulation. Eur J Pharm Biopharm.

[37] Yin S, Ji C, Wu P, Jin C, Qian H (2019). Human umbilical cord mesenchymal stem cells and exosomes: bioactive ways of tissue injury repair. Am J Transl Res.

[38] Xiong F, Ling X, Chen X, Chen J, Tan J, Cao W, Ge L, Ma M, Wu J. Pursuing specific chemotherapy of Orthotopic breast Cancer with lung metastasis from docking nanoparticles driven by bioinspired exosomes. Nano Lett. 2019. 10.1021/acs.nanolett.9b00824 30965009

[39] Jang YY, Collector MI, Baylin SB, Diehl AM, Sharkis SJ (2004). Hematopoietic stem cells convert into liver cells within days without fusion. Nat Cell Biol.

[40] Genschmer KR, Russell DW, Lal C, Szul T, Bratcher PE, Noerager BD, Abdul Roda M, Xu X, Rezonzew G, Viera L (2019). Activated PMN exosomes: pathogenic entities causing matrix destruction and disease in the lung. Cell.

[41] Chen LL, Yang L (2015). Gear up in circles. Mol Cell.

[42] Westholm JO, Miura P, Olson S, Shenker S, Joseph B, Sanfilippo P, Celniker SE, Graveley BR, Lai EC (2014). Genome-wide analysis of drosophila circular RNAs reveals their structural and sequence properties and age-dependent neural accumulation. Cell Rep.

[43] Guo JU, Agarwal V, Guo H, Bartel DP (2014). Expanded identification and characterization of mammalian circular RNAs. Genome Biol.

[44] Geng HH, Li R, Su YM, Xiao J, Pan M, Cai XX, Ji XP (2016). The circular RNA Cdr1as promotes myocardial infarction by mediating the regulation of miR-7a on its target genes expression. PLoS One.

[45] Burd CE, Jeck WR, Liu Y, Sanoff HK, Wang Z, Sharpless NE (2010). Expression of linear and novel circular forms of an INK4/ARF-associated non-coding RNA correlates with atherosclerosis risk. PLoS Genet.

[46] Du WW, Yang W, Chen Y, Wu ZK, Foster FS, Yang Z, Li X, Yang BB (2017). Foxo3 circular RNA promotes cardiac senescence by modulating multiple factors associated with stress and senescence responses. Eur Heart J.

[47] Wang K, Long B, Liu F, Wang JX, Liu CY, Zhao B, Zhou LY, Sun T, Wang M, Yu T (2016). A circular RNA protects the heart from pathological hypertrophy and heart failure by targeting miR-223. Eur Heart J.

[48] Li TR, Jia YJ, Wang Q, Shao XQ, Lv RJ (2017). Circular RNA: a new star in neurological diseases. Int J Neurosci.

[49] van Rossum D, Verheijen BM, Pasterkamp RJ (2016). Circular RNAs: novel regulators of neuronal development. Front Mol Neurosci.

[50] Zhang HD, Jiang LH, Sun DW, Hou JC, Ji ZL (2018). CircRNA: a novel type of biomarker for cancer. Breast Cancer.

[51] Yang W, Du WW, Li X, Yee AJ, Yang BB (2016). Foxo3 activity promoted by non-coding effects of circular RNA and Foxo3 pseudogene in the inhibition of tumor growth and angiogenesis. Oncogene.

[52] Guarnerio J, Bezzi M, Jeong JC, Paffenholz SV, Berry K, Naldini MM, Lo-Coco F, Tay Y, Beck AH, Pandolfi PP (2016). Oncogenic role of fusion-circRNAs derived from Cancer-associated chromosomal translocations. Cell.

[53] Bahn JH, Zhang Q, Li F, Chan TM, Lin X, Kim Y, Wong DT, Xiao X (2015). The landscape of microRNA, Piwi-interacting RNA, and circular RNA in human saliva. Clin Chem.

[54] Memczak S, Papavasileiou P, Peters O, Rajewsky N (2015). Identification and characterization of circular RNAs as a new class of putative biomarkers in human blood. PLoS One.

[55] Robbins PD, Morelli AE (2014). Regulation of immune responses by extracellular vesicles. Nat Rev Immunol.

[56] Turchinovich A, Weiz L, Burwinkel B (2012). Extracellular miRNAs: the mystery of their origin and function. Trends Biochem Sci.

[57] Clayton A, Mason MD (2009). Exosomes in tumour immunity. Curr Oncol.

[58] Li Y, Zheng Q, Bao C, Li S, Guo W, Zhao J, Chen D, Gu J, He X, Huang S (2015). Circular RNA is enriched and stable in exosomes: a promising biomarker for cancer diagnosis. Cell Res.

[59] Barbagallo C, Brex D, Caponnetto A, Cirnigliaro M, Scalia M, Magnano A, Caltabiano R, Barbagallo D, Biondi A, Cappellani A (2018). LncRNA UCA1, upregulated in CRC biopsies and downregulated in serum exosomes, controls mRNA expression by RNA-RNA interactions. Mol Ther Nucleic Acids.

[60] Dou Y, Cha DJ, Franklin JL, Higginbotham JN, Jeppesen DK, Weaver AM, Prasad N, Levy S, Coffey RJ, Patton JG, Zhang B (2016). Circular RNAs are down-regulated in KRAS mutant colon cancer cells and can be transferred to exosomes. Sci Rep.

[61] Li Z, Yanfang W, Li J, Jiang P, Peng T, Chen K, Zhao X, Zhang Y, Zhen P, Zhu J, Li X (2018). Tumor-released exosomal circular RNA PDE8A promotes invasive growth via the miR-338/MACC1/MET pathway in pancreatic cancer. Cancer Lett.

[62] Zhang Haiyang, Zhu Lei, Bai Ming, Liu Ying, Zhan Yang, Deng Ting, Yang Haiou, Sun Wu, Wang Xinyi, Zhu Kegan, Fan Qian, Li Jialu, Ying Guoguang, Ba Yi (2019). Exosomal circRNA derived from gastric tumor promotes white adipose browning by targeting the miR‐133/PRDM16 pathway. International Journal of Cancer.

[63] Zhang Haiyang, Deng Ting, Ge Shaohua, Liu Ying, Bai Ming, Zhu Kegan, Fan Qian, Li Jialu, Ning Tao, Tian Fei, Li Hongli, Sun Wu, Ying Guoguang, Ba Yi (2018). Exosome circRNA secreted from adipocytes promotes the growth of hepatocellular carcinoma by targeting deubiquitination-related USP7. Oncogene.

[64] Smyth SS, McEver RP, Weyrich AS, Morrell CN, Hoffman MR, Arepally GM, French PA, Dauerman HL, Becker RC, Platelet Colloquium P (2009). Platelet functions beyond hemostasis. J Thromb Haemost.

[65] French PA (2009). Platelet functions beyond hemostasis, antiplatelet therapies in high-risk patient subgroups: the fourth annual Platelet Colloquium. J Thromb Thrombolysis.

[66] Zhao RT, Zhou J, Dong XL, Bi CW, Jiang RC, Dong JF, Tian Y, Yuan HJ, Zhang JN (2018). Circular ribonucleic acid expression alteration in exosomes from the brain extracellular space after traumatic brain injury in mice. J Neurotrauma.

[67] Lobb RJ, Lima LG, Moller A (2017). Exosomes: key mediators of metastasis and pre-metastatic niche formation. Semin Cell Dev Biol.

[68] Zhang L, Zhang S, Yao J, Lowery FJ, Zhang Q, Huang WC, Li P, Li M, Wang X, Zhang C (2015). Microenvironment-induced PTEN loss by exosomal microRNA primes brain metastasis outgrowth. Nature.

[69] Whiteside TL (2017). The effect of tumor-derived exosomes on immune regulation and cancer immunotherapy. Future Oncol.

[70] Chen G, Huang AC, Zhang W, Zhang G, Wu M, Xu W, Yu Z, Yang J, Wang B, Sun H (2018). Exosomal PD-L1 contributes to immunosuppression and is associated with anti-PD-1 response. Nature.

[71] Fernandez PL, Hernandez L, Farre X, Campo E, Cardesa A (2002). Alterations of cell cycle-regulatory genes in prostate cancer. Pathobiology.

[72] Xu Y, Xia F, Ma L, Shan J, Shen J, Yang Z, Liu J, Cui Y, Bian X, Bie P, Qian C (2011). MicroRNA-122 sensitizes HCC cancer cells to adriamycin and vincristine through modulating expression of MDR and inducing cell cycle arrest. Cancer Lett.

[73] Fang F, Chang RM, Yu L, Lei X, Xiao S, Yang H, Yang LY (2015). MicroRNA-188-5p suppresses tumor cell proliferation and metastasis by directly targeting FGF5 in hepatocellular carcinoma. J Hepatol.

[74] Xue J, Liu Y, Luo F, Lu X, Xu H, Liu X, Lu L, Yang Q, Chen C, Fan W, Liu Q (1863). Circ100284, via miR-217 regulation of EZH2, is involved in the arsenite-accelerated cell cycle of human keratinocytes in carcinogenesis. Biochim Biophys Acta Mol basis Dis.

[75] Lu L, Luo F, Liu Y, Liu X, Shi L, Lu X, Liu Q (2015). Posttranscriptional silencing of the lncRNA MALAT1 by miR-217 inhibits the epithelial-mesenchymal transition via enhancer of zeste homolog 2 in the malignant transformation of HBE cells induced by cigarette smoke extract. Toxicol Appl Pharmacol.

[76] Chen DL, Zhang DS, Lu YX, Chen LZ, Zeng ZL, He MM, Wang FH, Li YH, Zhang HZ, Pelicano H (2015). microRNA-217 inhibits tumor progression and metastasis by downregulating EZH2 and predicts favorable prognosis in gastric cancer. Oncotarget.

[77] Zhang M, Li M, Li N, Zhang Z, Liu N, Han X, Liu Q, Liao C (2017). miR-217 suppresses proliferation, migration, and invasion promoting apoptosis via targeting MTDH in hepatocellular carcinoma. Oncol Rep.

[78] Wang X, Li M, Wang Z, Han S, Tang X, Ge Y, Zhou L, Zhou C, Yuan Q, Yang M (2015). Silencing of long noncoding RNA MALAT1 by miR-101 and miR-217 inhibits proliferation, migration, and invasion of esophageal squamous cell carcinoma cells. J Biol Chem.

[79] Bachmann IM, Halvorsen OJ, Collett K, Stefansson IM, Straume O, Haukaas SA, Salvesen HB, Otte AP, Akslen LA (2006). EZH2 expression is associated with high proliferation rate and aggressive tumor subgroups in cutaneous melanoma and cancers of the endometrium, prostate, and breast. J Clin Oncol.

[80] Aoki R, Chiba T, Miyagi S, Negishi M, Konuma T, Taniguchi H, Ogawa M, Yokosuka O, Iwama A (2010). The polycomb group gene product Ezh2 regulates proliferation and differentiation of murine hepatic stem/progenitor cells. J Hepatol.

[81] Lee Y, Dominy JE, Choi YJ, Jurczak M, Tolliday N, Camporez JP, Chim H, Lim JH, Ruan HB, Yang X (2014). Cyclin D1-Cdk4 controls glucose metabolism independently of cell cycle progression. Nature.

[82] Tian L, Cao J, Jiao H, Zhang J, Ren X, Liu X, Liu M, Sun Y (2019). CircRASSF2 promotes laryngeal squamous cell carcinoma progression by regulating the miR-302b-3p/IGF-1R axis. Clin Sci (Lond).

[83] Fidler IJ (2003). The pathogenesis of cancer metastasis: the 'seed and soil' hypothesis revisited. Nat Rev Cancer.

[84] Luna J, Boni J, Cuatrecasas M, Bofill-De Ros X, Nunez-Manchon E, Gironella M, Vaquero EC, Arbones ML, de la Luna S, Fillat C. DYRK1A modulates c-MET in pancreatic ductal adenocarcinoma to drive tumour growth. Gut. 2018;0:1–12. 10.1136/gutjnl-2018-316128 10.1136/gutjnl-2018-316128 30343272

[85] Zhang X, Wang S, Wang H, Cao J, Huang X, Chen Z, Xu P, Sun G, Xu J, Lv J, Xu Z (2019). Circular RNA circNRIP1 acts as a microRNA-149-5p sponge to promote gastric cancer progression via the AKT1/mTOR pathway. Mol Cancer.

[86] Wang J, Zhang Q, Zhou S, Xu H, Wang D, Feng J, Zhao J, Zhong S (2019). Circular RNA expression in exosomes derived from breast cancer cells and patients. Epigenomics.

[87] Wang Guoying, Liu Wei, Zou Yong, Wang Genshu, Deng Yinan, Luo Jingyan, Zhang Yingcai, Li Hua, Zhang Qi, Yang Yang, Chen Guihua (2019). Three isoforms of exosomal circPTGR1 promote hepatocellular carcinoma metastasis via the miR449a–MET pathway. EBioMedicine.

[88] Reymond N, d’Agua BB, Ridley AJ (2013). Crossing the endothelial barrier during metastasis. Nat Rev Cancer.

[89] Schaeffer RC, Gong F, Bitrick MS, Smith TL (1993). Thrombin and bradykinin initiate discrete endothelial solute permeability mechanisms. Am J Phys.

[90] Lum H, Malik AB (1996). Mechanisms of increased endothelial permeability. Can J Physiol Pharmacol.

[91] Garcia JG, Verin AD, Schaphorst KL (1996). Regulation of thrombin-mediated endothelial cell contraction and permeability. Semin Thromb Hemost.

[92] Wang SC, Lin XL, Li J, Zhang TT, Wang HY, Shi JW, Yang S, Zhao WT, Xie RY, Wei F (2014). MicroRNA-122 triggers mesenchymal-epithelial transition and suppresses hepatocellular carcinoma cell motility and invasion by targeting RhoA. PLoS One.

[93] Schlegel N, Baumer Y, Drenckhahn D, Waschke J (2009). Lipopolysaccharide-induced endothelial barrier breakdown is cyclic adenosine monophosphate dependent in vivo and in vitro. Crit Care Med.

[94] Wojciak-Stothard B, Potempa S, Eichholtz T, Ridley AJ (2001). Rho and Rac but not Cdc42 regulate endothelial cell permeability. J Cell Sci.

[95] van Nieuw Amerongen GP, van Hinsbergh VW (2007). Endogenous RhoA inhibitor protects endothelial barrier. Circ Res.

[96] Breslin JW, Yuan SY (2004). Involvement of RhoA and rho kinase in neutrophil-stimulated endothelial hyperpermeability. Am J Physiol Heart Circ Physiol.

[97] Lu Q, Lu L, Chen W, Chen H, Xu X, Zheng Z (2015). RhoA/mDia-1/profilin-1 signaling targets microvascular endothelial dysfunction in diabetic retinopathy. Graefes Arch Clin Exp Ophthalmol.

[98] Gorovoy M, Neamu R, Niu J, Vogel S, Predescu D, Miyoshi J, Takai Y, Kini V, Mehta D, Malik AB, Voyno-Yasenetskaya T (2007). RhoGDI-1 modulation of the activity of monomeric RhoGTPase RhoA regulates endothelial barrier function in mouse lungs. Circ Res.

[99] van Nieuw Amerongen GP, Draijer R, Vermeer MA, van Hinsbergh VW (1998). Transient and prolonged increase in endothelial permeability induced by histamine and thrombin: role of protein kinases, calcium, and RhoA. Circ Res.

[100] Chen X, Chen RX, Wei WS, Li YH, Feng ZH, Tan L, Chen JW, Yuan GJ, Chen SL, Guo SJ (2018). PRMT5 circular RNA promotes metastasis of urothelial carcinoma of the bladder through sponging miR-30c to induce epithelial-mesenchymal transition. Clin Cancer Res.

[101] Bach DH, Hong JY, Park HJ, Lee SK (2017). The role of exosomes and miRNAs in drug-resistance of cancer cells. Int J Cancer.

[102] Aldinucci D, Celegato M, Casagrande N (2016). Microenvironmental interactions in classical Hodgkin lymphoma and their role in promoting tumor growth, immune escape and drug resistance. Cancer Lett.

[103] Corcoran C, Rani S, O’Brien K, O’Neill A, Prencipe M, Sheikh R, Webb G, McDermott R, Watson W, Crown J, O’Driscoll L (2012). Docetaxel-resistance in prostate cancer: evaluating associated phenotypic changes and potential for resistance transfer via exosomes. PLoS One.

[104] Feng W, Su Z, Yin Q, Zong W, Shen X, Ju S. ncRNAs associated with drug resistance and the therapy of digestive system neoplasms. J Cell Physiol. 2019;1-15. 10.1002/jcp.28551 10.1002/jcp.28551 30941775

[105] Li L, Li W, Chen N, Zhao H, Xu G, Zhao Y, Pan X, Zhang X, Zhou L, Yu D (2019). FLI1 Exonic circular RNAs as a novel oncogenic driver to promote tumor metastasis in small cell lung Cancer. Clin Cancer Res.

[106] Andreyev HJ, Norman AR, Cunningham D, Oates J, Dix BR, Iacopetta BJ, Young J, Walsh T, Ward R, Hawkins N (2001). Kirsten ras mutations in patients with colorectal cancer: the ‘RASCAL II’ study. Br J Cancer.

[107] Seimiya T, Otsuka M, Liu H-Y, Suzuki T, Sekiba K, Yamagami M, Tanaka E, Ishibashi R, Koike K (2018). Circular RNA and exosomes in pancreatic cancer progression. Transl Cancer Res.

[108] Duijvesz D, Burnum-Johnson KE, Gritsenko MA, Hoogland AM, Vredenbregt-van den Berg MS, Willemsen R, Luider T, Pasa-Tolic L, Jenster G (2013). Proteomic profiling of exosomes leads to the identification of novel biomarkers for prostate cancer. PLoS One.

[109] Zhou L, Lv T, Zhang Q, Zhu Q, Zhan P, Zhu S, Zhang J, Song Y (2017). The biology, function and clinical implications of exosomes in lung cancer. Cancer Lett.

[110] Peinado H, Alec Kovic M, Lavotshkin S, Matei I, Costa-Silva B, Moreno-Bueno G, Hergueta-Redondo M, Williams C, Garcia-Santos G, Ghajar CM (2016). Corrigendum: melanoma exosomes educate bone marrow progenitor cells toward a pro-metastatic phenotype through MET. Nat Med.

[111] Kristensen LS, Hansen TB, Veno MT, Kjems J (2018). Circular RNAs in cancer: opportunities and challenges in the field. Oncogene.

